# Oxidative Hemolysis of Erythrocytes Induced by Various Vitamins

**Published:** 2006-09

**Authors:** I. H. Ibrahim, S. M. Sallam, H. Omar, M. Rizk

**Affiliations:** 1 *Department of Physics, Faculty of Science, Ain Shams University, Cairo. Egypt;*; 2 *Department of Physics, Faculty of Science, Benha University, Benha. Egypt*

**Keywords:** hemolysis, erythrocytes, niacin, pyridoxine, thiamine, ascorbic acid, conductivity

## Abstract

Hemolytic effect of some water-soluble vitamins (niacin B_5_, pyridoxine B_6_, thiamine B_1_ and ascorbic and acid C) on erythrocytes was studied spectrophotometrically at relatively high concentration. The oxidation mechanism of hemoglobin was the same for the used vitamins. Vitamin C was the strongest hemolytic agent in comparison with the other vitamins, while vitamin B_1_ is the weakest one. The results were confirmed by studying the variation in conductivity of erythrocytes with temperature in the range 20-40°C for the used vitamins at a concentration of 2 mM and after two hours from adding each vitamin to the erythrocytes suspension. The conductivity measurements show that the conductivity for the used vitamins is lower than that for control (without adding vitamin) due to hemoglobin oxidation, also may be due to the electrical reorganization of the erythrocyte membrane after the interaction of the used vitamin with it. The obtained results insure the oxidizing effect of the used vitamins on hemoglobin and consequently their hemolytic effect on erythrocytes.

## INTRODUCTION

Oxidative damage to biological systems is the basis of a number of physiological and pathological phenomena (1). For that it has been presented in great number of studies. Erythrocytes have often been used as a convenient model for these studies. The oxidation process in erythrocytes affects the over all cell structures, hemoglobin and membrane. Several hypotheses have been proposed to explain the mechanism of erythrocytes hemolysis following oxidative stress *in vivo* and *in vitro* (2). Hemoglobin appears to be the main site of damage when various oxidative drugs are used (3). Under other oxidative conditions, the membrane appears to be the target of injury leading to hemolysis (4).

Oxidative denaturation of hemoglobin is a process in which oxidative changes in the molecule cause its disruption. This process takes place when the concentration of oxidants in a red cell is increased. The intracellular aggregates of denatured hemoglobin produced when oxidative denaturation occurs in the red cell are called Heinz bodies (5). The formation of Heinz bodies is directly associated with the induction of hemolytic anemia. Many chemical compounds cause the oxidation of hemoglobin, yet underlying molecular mechanisms remain unknown. Many vitamins are commonly believed to be nontoxic although toxicity can appear when large amounts of these vitamins are consumed (6). Several authors have developed different methods to measure the antioxidant activity of many compounds: pulse radiolysis (7), inhibition of the oxidation of chemical and biological targets (8, 9). Among them, the hemolysis assays have already been used for a long time in measuring free radical damages. Previous studies (10-12) have demonstrated the hemolytic effect of ascorbic acid (Vitamin C) on erythrocytes and they attributed it to an oxidative damage (oxidative hemolysis).

The study of the passive electrical properties of the cell membrane is an area of active interest, yielding a lot of information on the structure and physiology of cells and different cell compartments (13). The aim of the present work is to study the hemolytic effect of some water-soluble vitamins (niacin B_5_, pyridoxine B_6_, thiamine B_1_ and ascorbic acid C) on erythrocytes when added with relatively high concentration.

## MATERIALS AND METHODS

### Preparation of erythrocytes suspension

Human blood from healthy donors, ant coagulated with heparin, was stored at 4°C and used for experiment in the same day. Erythrocytes were isolated by centrifugation (10 min, 1500 revolution/min), plasma and buffy coat removed and the cells washed three times with isotonic saline (0.15 M NaCl) at room temperature. Washed erythrocytes were suspended in saline to reach a concentration of about 3 × 10^5^ cells/ml.

### Registration of hemolysis

Hemolysis or kinetics of hemoglobin breakdown in erythrocytes exposed to the used vitamins with different concentrations [B_5_ (0.25, 0.5, 1.0, and 2.0 mM), B_6_ (1.0, 2.0, 4.0, and 8.0 mM), B_1_ (0.25, 0.5, 1.0 and 2.0 mM) and C (0.5, 0.8, 2.0 and 4.0 mM)] were registered spectrophotometrically (Jenway spectrophotometer model 6300) at 577 nm (spin state band of iron- hem), where the decrease in the intensity of the peak at 577 nm represents the degree of hemoglobin breakdown or degree of hemolysis. The cuvette of the spectrophotometer was filled by 5.8 ml of the suspension and 0.2ml of the used vitamin of certain concentration, then the value of the absorbance at 577nm was recorded after shaking gently and carefully before each measure.

In this part, the measured values of absorbance are a representative experiment and error bars cannot be calculated on raw absorbance values due to day to day variations in total spectrometric readings.

### Conductivity measurements

The used apparatus was a conductance bridge (Griffen and George Ltd. London) with glass tube filled by the suspension, containing two platinum disc electrodes separated by a fixed distance. For recording the variations in conductivity with temperature, 0.2 ml of the used concentration of vitamin was added to 5.8 ml of erythrocytes suspension and the mixture was left 2 hours till complete hemolysis is fulfilled, then the variation in conductivity with temperature is recorded, by warming the glass tube holding the mixture in a water bath.

## RESULTS AND DISCUSSION

**Figure 1:**
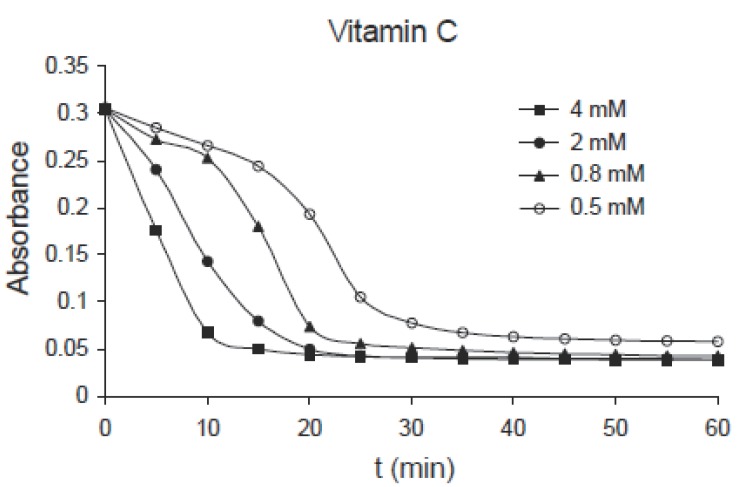
Kinetics of hemoglobin breakdown in erythrocytes exposed to different concentrations of ascorbic acid.

**Figure 2:**
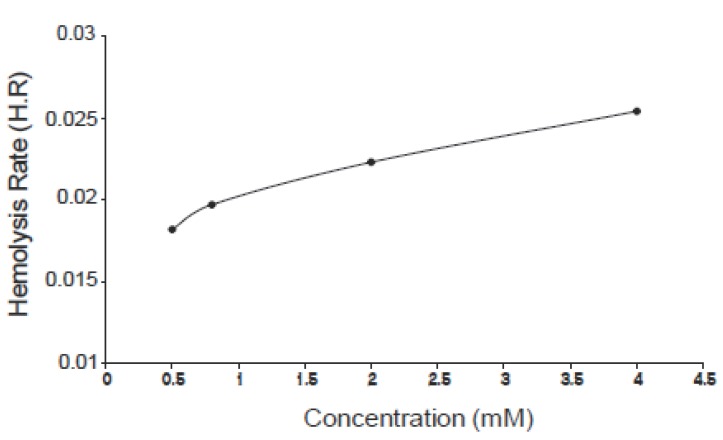
Variation of the hemolysis rate (H.R) with concentration of vitamin C.

The kinetics of hemoglobin breakdown in erythrocytes exposed to different concentrations of ascorbic acid (0.5, 0.8, 2.0 and 4.0 mM) is shown in Fig. 1, in which the absorbance decreases with time for each concentration, this means that the degree of hemolysis (degree of hemoglobin breakdown) increases with time. It was shown also that the degree of hemolysis depends on the concentration of ascorbic acid, in which it increases by increasing concentration. From this figure there is a time interval during which the variations in absorbance with time is nearly linear, the slope of this interval gives the hemolysis rate (H.R); the rate at which the number of cells in the suspension decreases. Fig. 2 shows the variation of hemolysis rate (H.R) with different concentrations of vitamin C. From this figure it is clear that the hemolysis rate increases by increasing the concentration. The obtained results indicate that the number of erythrocytes decreases rapidly at higher concentrations (hemolysis). The hemolysis was believed to be due to the interaction of ascorbic acid with erythrocytes causing lipid peroxidation of membrane and oxidation of hemoglobin (oxidation of Fe^+2^ to Fe^+3^) (10-12). This process leads to hemolysis of erythrocytes if the concentration of ascorbic acid is relatively high (low concentrations of ascorbic acid act as antioxidants).

**Figure 3:**
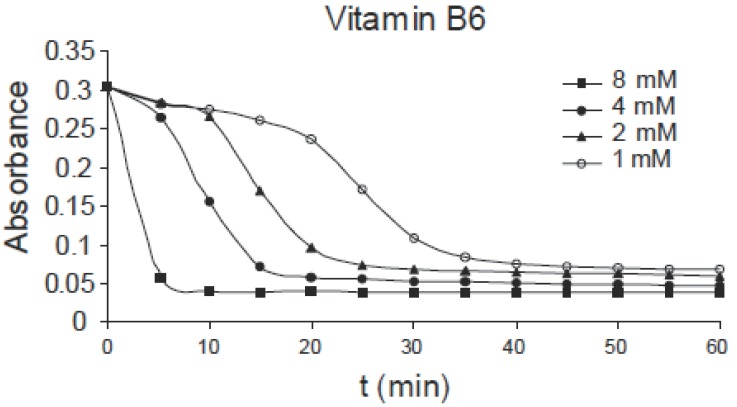
Absorbance variation of erythrocytes with time due to the effect of different concentrations of B_6_.

**Figure 4:**
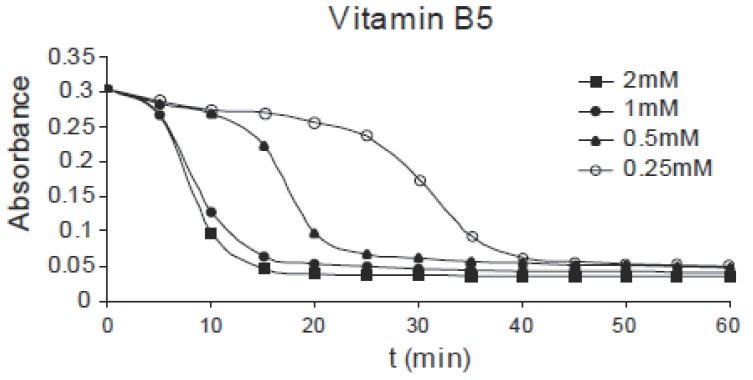
Absorbance variation of erythrocytes with time due to the effect of different concentrations of B_5_.

**Figure 5:**
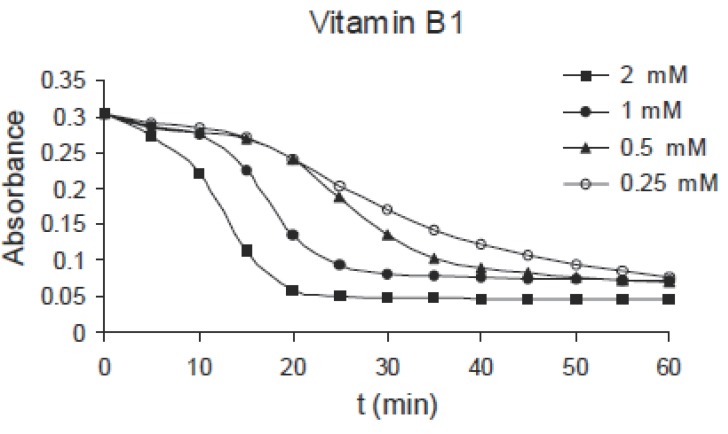
Absorbance variation of erythrocytes with time due to the effect of different concentrations of B_1_.

**Figure 6:**
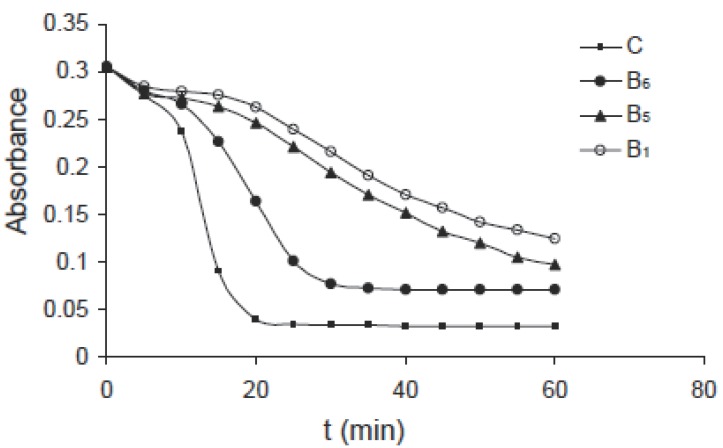
Kinetics of hemoglobin breakdown in erythrocytes due to the effect of the used vitamins at concentration of 2 mM.

The same effect can be observed for pyridoxine B_6_ of concentrations: 1.0, 2.0, 4.0 and 8.0 mM as shown in Fig. 3, for niacin B_5_ of concentrations: 0.25, 0.5, 1.0 and 2.0 mM as shown in Fig. 4 and for thiamine B_1_ of concentrations: 0.25, 0.5, 1.0 and 2.0 mM as shown in Fig. 5. Comparing the four figures, it may be concluded that the mechanism of hemoglobin breakdown and consequently hemolysis of erythrocytes is the same for the four used vitamins and as described before for ascorbic acid. Fig. 6 shows the effect of the four vitamins at concentration of 2mM on erythrocytes suspension. From figure it is clear that vitamin C is the strongest hemolytic agent in comparison with the other vitamins, in which the hemolysis is fulfilled at about 20 minutes from adding the used concentration (2.0 mM), while vitamin B_1_ is the weakest one.

**Figure 7:**
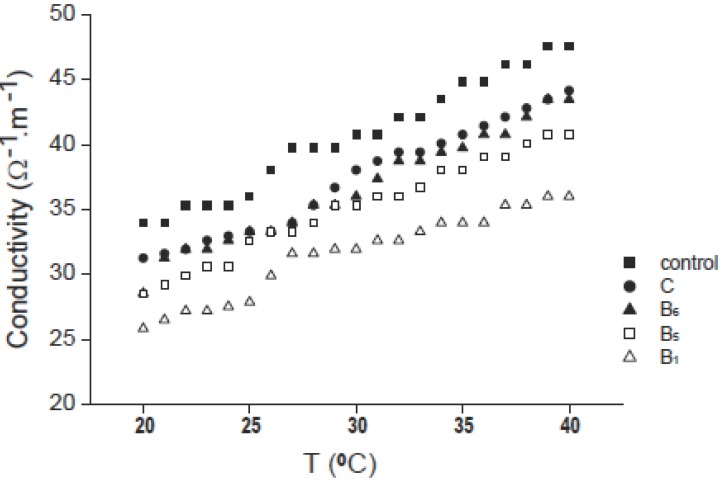
Variation of conductivity with temperature for control and vitamins C, B_6_, B_5_ and B_1_ at concentration of 2 mM.

The variation in conductivity of erythrocytes with temperature in the range 20-40°C is shown in Fig. 7 for control (without adding vitamin) and for the four used vitamins at a concentration of 2 mM and after two hours from adding vitamin. From figure it is clear that the conductivity for the used vitamins is lower than that for control due to hemoglobin oxidation, also for all curves the conductivity increases by increasing temperature. The figure also shows that the conductivity of vitamin C is the higher one in comparison with other vitamins. Vitamins C and B_6_ are known to cross cell membranes easily and to be taken into the red blood cell, oxidizing hemoglobin to methemoglobin (14), so decreases the conductivity of the suspension (12). Also, the change in electrical conductivity due to the addition of vitamin in comparison with control may be due to the electrical re-organization of the erythrocyte membrane after the interaction of such vitamin with it (15). The difference in the values of conductivity- after adding the used vitamins to erythrocytes suspensions-from one vitamin to another maybe due to the difference in conductivity of such vitamins themselves.

The obtained results insure the oxidizing effect of the used vitamins on hemoglobin and consequently their hemolytic effect on erythrocytes.
